# The Importance of Safely Prescribing Hormones in the Transgender Community

**DOI:** 10.7759/cureus.44639

**Published:** 2023-09-04

**Authors:** Christopher J Vaccaro, Sergio A Karageuzian, Erin McFadden

**Affiliations:** 1 School of Osteopathic Medicine, A.T. Still University of Health Sciences, Mesa, USA; 2 Internal Medicine, The Wright Center for Graduate Medical Education, Scranton, USA

**Keywords:** hormone replacement therapy, pregnancy care, modern medicine, pregnancy counseling, transgender health

## Abstract

The transgender community is rapidly growing, necessitating further education and understanding of their unique healthcare needs. Gender affirmation is a multistep process, which generally begins with transgender individuals socially transitioning by adopting a new name, pronouns, and appearance changes, such as hair and clothing, to express themselves. Additional gender affirmation treatment can be achieved through medical therapy with hormones and surgical intervention.

Here, we report the case of an 18-year-old transgender man who presented to his primary care provider for a referral to initiate a medical transition with testosterone therapy. The patient practiced penetrative vaginal sex without contraception. The patient presented to the clinic eight months later with amenorrhea, thick coarse facial and body hair, oily skin, irritable moods, and a 20-lb weight gain. A pregnancy test revealed a positive result. A healthy baby girl was delivered at 40 weeks’ gestation.

This case demonstrates the importance of addressing contraception during the masculinization process in individuals who continue to practice vaginal intercourse. Healthcare providers should seek to establish a clinical environment free of discrimination and stigma to allow patients to feel comfortable describing potential triggers of gender dysphoria. We encourage medical practitioners to discuss all methods of birth control with transgender male patients and choose the contraceptive that best allows for a seamless medical transition.

## Introduction

With each successive generation, transgender and gender nonconformity communities have gained increasing acceptance in society. Approximately 1.6% of adults in the United States identify as transgender or nonbinary, a number that more than triples to 5.1% when discussing adults under the age of 30 years [1]. The growing population of transgender and gender-nonconforming individuals necessitates the need for medical providers to develop cultural competence to better care for these communities. These young people have various lifestyles and practices that require providers to build rapport to deliver the best care possible. Such patients engage in various sexual practices, each of which confers different risks. Moreover, 39% of trans men engage in intravaginal sex, and vaginal intercourse can result in the transmission of sexually transmitted diseases and pregnancy [2].

Transgender and nonconforming individuals may seek hormone therapy to live according to their gender identity, while socially transitioned individuals may desire further physical features that are congruent with their ideal body. According to Grant et al., 66% of female-to-male transgender individuals seek hormonal therapy [3]. Testosterone is a steroid hormone that promotes masculine body features. The current guidelines for medical gender transition are put forth by the World Professional Association for Transgender Health (WPATH) and the Endocrine Society to aid healthcare providers. Eligible patients must have had a marked, sustained gender incongruence experience, together with stable mental health and the ability to give informed consent [4,5]. Healthcare providers are highly recommended to obtain a full sexual history and screen for pre-existing conditions that may worsen during testosterone therapy [6]. Testosterone therapy is contraindicated in pregnancy and in people seeking to become pregnant [7]. Providers are recommended to follow patients on hormone therapy every three months to assess virilization and response to treatment. Testosterone effects can be observed in the first three to six months. Patients can expect to experience increased body and facial hair, amenorrhea, increased acne and skin changes, increased muscle mass, and increased libido [8]. Over the following one to two years, patients can develop varying degrees of a deepened voice, vaginal atrophy, clitoral enlargement, and male pattern baldness [8]. Although testosterone decreases gender dysphoria, patients should be aware of some documented adverse effects such as social distress, anxiety, and depression [9]. Additionally, specific consideration must be given to people with coronary artery disease, hypertension, dyslipidemia, and people considering pregnancy.

Testosterone does not prevent pregnancy, and physicians should discuss contraception to prevent unwanted pregnancy. Testosterone acts on peripheral tissues to increase the previously stated effects. Testosterone is theorized to suppress menstruation by inactivating endometrial tissue [10]. However, as the female reproductive system is not completely suppressed by testosterone, ovulation is still possible [11]. This emphasizes the need for trans men to use some form of contraception during the transition process if they practice penetrative vaginal sex. Patients should be counseled regarding the possibility of pregnancy while on testosterone therapy and provided with effective birth control options if desired.

Counseling patients on birth control during the gender transition process is strongly recommended by WPATH [4]. Studies have shown that 16%-31% of transgender men believe that testosterone is a form of contraception [12-14]. Healthcare providers are also misinformed regarding the effects of testosterone, and approximately 5.5%-9% of transgender men have been advised of the contraceptive effects of testosterone by physicians [12,14]. Physicians should seek regular updates regarding the reproductive health of transgender persons to best advise them in their gender transition journey. Physicians and patients should have a genuine discussion regarding sex practices and reproductive education, with the aim of initiating safe birth control methods to allow for an ideal masculinization transition process.

Testosterone plays a critical role in fetal development and aids in the maturation of the central nervous and genitourinary systems. During pregnancy, testosterone is produced in the fetal-placental unit, maternal ovaries, and adrenals, with variable involvement of peripheral adipose tissue [15]. Throughout pregnancy, the physiologic levels of testosterone vary greatly and are estimated to be approximately 26-211 ng/dL in the first trimester, 34-243 ng/dL in the second trimester, and 63-309 ng/dL in the third trimester [16]. However, previous studies have been unable to identify a definitive correlation between maternal-fetal testosterone levels [17-19]. One potential explanation is the action of placental aromatase, which can be protective against direct maternal testosterone transfer [20]. Excessive prenatal androgens can result in the virilization of female genitalia [21]. Few case studies have examined the effects of increased prenatal androgen exposure from conception to late pregnancy, labor, and delivery. However, in a twin study with maternal exposure to topical exogenous testosterone, twin A (female) exhibited clitoromegaly and Prader Stage 2 of genitalia, whereas twin B (female) exhibited normal genitalia [22]. Additional research has shown that elevated maternal testosterone is associated with intrauterine growth restriction and preeclampsia risk [23-26]. Moreover, testosterone-induced vaginal atrophy can cause protraction of labor, potentially necessitating operative intervention (forceps, vacuum, cesarean) or complications such as vaginal tears [27].

## Case presentation

Here, we present the case of an 18-year-old transgender man (pronouns they/them, he/him), who, accompanied by his mother, presented to our clinic in February 2022 seeking to establish care, primarily a referral to endocrinology for hormonal therapy. The patient wished to have more masculine features to match his gender identity and improve his mental health. The patient reported regular menstruation every month. The patient’s medical history included gender dysphoria, depression, attention-deficit/hyperactivity disorder, and idiopathic urticaria. The patient attended weekly therapy sessions with a psychiatrist but took no medications and denied suicidal or homicidal ideations. The patient’s mother had depression and the father had autism. The patient was assigned female at birth but socially transitioned to a man in the ninth grade and responded to a chosen male name. The patient was bisexual and denied undergoing gender-affirming surgery.

The patient’s vitals were as follows: blood pressure, 117/78 mmHg; heart rate, 77 beats per minute; temperature, 97.6°F; respirations, 16 breaths per minute; weight, 101 lb (48.814 kg); height, 62 inches; and body mass index (BMI), 18.5 kg/m^2^.

The patient’s weight was within the sixth percentile, and their BMI was within the 13th percentile. Physical examination revealed a pleasant, cooperative, thin, petite individual with feminine characteristics, short black hair, and loose clothing, with no abnormal findings identified.

The patient was evaluated by an endocrinologist one month later. The patient had extensive discussions regarding the risks and benefits of testosterone therapy. The patient was explicitly warned about the possibility of infertility. He conveyed his understanding and expressed no desire to have future biological children. A letter of support was received from the patient’s mental health provider, and baseline laboratory findings were within normal limits. As a result, the endocrinologist initiated transdermal androgel 1.62%, two pumps daily.

In May 2022, the patient was seen by his primary care provider. The patient tolerated hormone replacement well and had developed appropriate testosterone-induced phenotypes, including thick coarse facial and body hair, oily skin, and irritable moods. The patient’s last menstrual period had occurred at the end of April 2022. At a six-month follow-up with his primary care provider in November 2022, the patient complained of episodic palpitations, lightheadedness, and diffuse joint pain. The patient had also gained 19 lb (8.6 kg).

The patient’s grandmother presented to the primary care clinic two weeks later for a routine well visit. At that time, she informed the primary care physician that her trans grandson (patient above) was pregnant. The patient was seen in the emergency department where an ultrasound revealed a single live intrauterine pregnancy dated at 33 weeks and five days, with characteristics associated with a female fetus. The primary care provider immediately contacted the patient with instructions to discontinue testosterone therapy.

At 40 weeks’ gestation, a healthy baby girl was delivered by normal spontaneous vaginal delivery. Physical examination showed normal external female genitalia, with no abnormal findings. The baby’s vitals were as follows: height, 48.5 cm; weight, 3.34 kg; and APGAR, 8 and 9 at one and five minutes, respectively. The newborn child is doing well, established with a pediatrician, and will be closely monitored going forward.

## Discussion

This case highlights the importance of the physician-patient relationship. The development of a mutual sense of understanding between the patient and healthcare providers is crucial. A conversation regarding sexual practices is mandatory and cannot be overlooked. Trust must be established to have these sensitive conversations. In this case, the patient did not feel comfortable disclosing his sexual practices; when the conversation did occur, he denied having intravaginal sex. Such apprehension and distrust between the transgender community and the medical community is common. Indeed, according to Lerner et al., in 2021, 22% of transgender, gender-nonconforming, nonbinary people avoided seeking medical care due to anticipated disrespect or mistreatment when visiting a physician [28]. Many studies have highlighted possible reasons for this, including, but not limited to, misgendering, perceived discriminatory behavior, verbal abuse, and physical abuse [28-30]. Worse still, 50% of transgender patients reported having to teach their medical providers about transgender care [31]. Physicians often lack appropriate education and cultural competency regarding transgender populations [32,33]. In this case, without the establishment of an authentic physician relationship, discussion regarding contraception never occurred. As such, when symptoms of rapid weight gain, amenorrhea, and abdominal discomfort manifested, they were attributed to hormonal therapy. Thus, the fetus was continuously exposed to exogenous testosterone until 33 weeks’ gestation. Healthcare providers should be aware that the transgender and nonbinary communities can feel victimized by the medical community. If mutual trust had been established in this case, perhaps the more accepting clinical environment would have facilitated the patient to disclose their sexual practices, allowing contraception to have been initiated.

Concurrent testosterone therapy is not a contraindication for any current birth control; therefore, all birth control should be offered to patients based on their individualized needs. Physicians should understand the specific triggers of gender dysphoria of trans men to initiate an appropriate form of birth control. Combined oral contraceptive pills (COCPs) are safe and effective while on testosterone therapy [11]. Studies have shown no increased venous thromboembolism risk compared to cisgender females [34,35]. However, as COCPs contain estrogen, which is traditionally viewed as feminine, taking a “feminine” pill every day can serve as a daily reminder of their previous gender identity [12]. Estrogen can also induce breast tenderness, which can exacerbate feelings of gender dysphoria [36]. Indeed, patients have reported a perceived decrease in the masculinization effects of testosterone while on COCPs [11]. Intrauterine devices (IUDs) and vaginal rings require a pelvic examination to place the device, which may cause emotional distress [37]. IUDs are a highly effective method of contraception, which can last for many years. Copper IUDs are favorable because they do not secrete hormones; however, they have been associated with dysmenorrhea and menorrhagia [38]. Progesterone IUDs can work synergistically with testosterone therapy to promote masculinization [11]. Subdermal progesterone-containing implants have the same effects but do not necessitate a potentially emotionally traumatic pelvic examination [39]. Irreversible sterilization procedures such as bilateral tubal ligation and hysterectomy are available but carry surgical and anesthesia risks. Physicians should acknowledge and understand potential triggers of gender dysphoria in trans men to initiate appropriate contraception.

## Conclusions

We report the case of an 18-year-old transgender man who practiced penetrative intravaginal sex and became pregnant, resulting in the accidental exposure of the growing fetus to exogenous testosterone. As the transgender community gains recognition in society, more individuals are presenting to healthcare providers seeking information regarding hormonal therapy. Healthcare providers should familiarize themselves with guidelines and seek education to better care for their transgender patients. This case depicts the need for healthcare delivery systems to evolve to meet the unique needs of the transgender community. Patients and healthcare providers should work together to break through stereotypical societal gender ideologies to better understand the roots of gender dysphoria and implement a safe form of birth control. The most appropriate birth control is patient-specific and allows for a safe and sensitive gender transition.
